# Derivation and internal validation of a data-driven prediction model to guide frontline health workers in triaging children under-five in Nairobi, Kenya

**DOI:** 10.12688/wellcomeopenres.15387.3

**Published:** 2021-04-19

**Authors:** Alishah Mawji, Samuel Akech, Paul Mwaniki, Dustin Dunsmuir, Jeffrey Bone, Matthew O. Wiens, Matthias Görges, David Kimutai, Niranjan Kissoon, Mike English, Mark J. Ansermino

**Affiliations:** 1 Department of Anesthesiology, Pharmacology & Therapeutics, University of British Columbia, Vancouver, British Columbia, V6T1Z3, Canada; 2 Centre for International Child Health, BC Children’s Hospital Research Institute, Vancouver, British Columbia, V5Z4H4, Canada; 3 Kenya Medical Research Institute/Wellcome Trust Research Programme, Nairobi, Kenya; 4 Digital Health Innovation Lab, BC Children’s Hospital Research Institute, Vancouver, British Columbia, V5Z4H4, Canada; 5 Department of Obstetrics and Gynaecology, University of British Columbia, Vancouver, British Columbia, V6T1Z4, Canada; 6 Mbagathi County Referral Hospital, Nairobi, Kenya; 7 Department of Pediatrics, University of British Columbia, Vancouver, British Columbia, V6H3V4, Canada; 8 Centre for Tropical Medicine and Global Health, Nuffield Department of Clinical Medicine, University of Oxford, Oxford, UK

**Keywords:** sepsis, prediction, risk, model, triage, children, developing countries

## Abstract

**Background: **Many hospitalized children in developing countries die from infectious diseases. Early recognition of those who are critically ill coupled with timely treatment can prevent many deaths. A data-driven, electronic triage system to assist frontline health workers in categorizing illness severity is lacking. This study aimed to develop a data-driven parsimonious triage algorithm for children under five years of age.

**Methods: **This was a prospective observational study of children under-five years of age presenting to the outpatient department of Mbagathi Hospital in Nairobi, Kenya between January and June 2018. A study nurse examined participants and recorded history and clinical signs and symptoms using a mobile device with an attached low-cost pulse oximeter sensor. The need for hospital admission was determined independently by the facility clinician and used as the primary outcome in a logistic predictive model. We focused on the selection of variables that could be quickly and easily assessed by low skilled health workers.

**Results: **The admission rate (for more than 24 hours) was 12% (N=138/1,132). We identified an eight-predictor logistic regression model including continuous variables of weight, mid-upper arm circumference, temperature, pulse rate, and transformed oxygen saturation, combined with dichotomous signs of difficulty breathing, lethargy, and inability to drink or breastfeed. This model predicts overnight hospital admission with an area under the receiver operating characteristic curve of 0.88 (95% CI 0.82 to 0.94). Low- and high-risk thresholds of 5% and 25%, respectively were selected to categorize participants into three triage groups for implementation.

**Conclusion: **A logistic regression model comprised of eight easily understood variables may be useful for triage of children under the age of five based on the probability of need for admission. This model could be used by frontline workers with limited skills in assessing children. External validation is needed before adoption in clinical practice.

## Introduction

Infectious diseases contribute to most deaths of children under five worldwide ^1^. Sub-Saharan Africa has the highest under-five mortality rate in the world, with one child in 13 dying before his or her fifth birthday ^1^. Death from infectious diseases is commonly due to a shared final pathway: sepsis, a dysregulated immune response leading to multi-organ dysfunction ^2^. Sepsis mortality rates in Africa are eight times higher than North America ^3^.

More than half the cases of infectious disease-related child mortality are preventable through prompt diagnosis and early initiation of emergency treatment ^1^. Triage, the practice of prioritizing patients for treatment based on the severity of illness, is critical to ensuring timely treatment ^4^. Triage systems in low-income settings continue to face challenges including limited numbers of expert clinicians and lack of adequately trained health workers ^4, 5^.

The World Health Organization (WHO) advocates the use of the Emergency Triage Assessment and Treatment (ETAT) guidelines to triage sick children in resource limited settings. The ETAT system is widely adopted in low- and middle-income countries (LMICs), where effective implementation has seen reductions in inpatient child mortality rates in Malawi and Sierra Leone ^6, 7^. However, ETAT relies on training, memorization, and clinical competence of the triage examiner rendering implementation difficult, and uptake uneven in many LMICs ^5, 8, 9^. ETAT is based on clinical decision rules which may limit generalizability, while the manual mechanisms of implementation provide little opportunity for monitoring and feedback, and there is limited ability to update it 10. Additionally, at hospitals affected by staff shortages, wait times to a formal ETAT triage are lengthy (can take multiple hours). 

One solution to these shortcomings is the use of a digital, data-driven approach to strengthen triage systems at first contact. Digital health platforms can facilitate quality improvement, while data-driven algorithms are easily updateable with emergence of new information and can be optimized to meet the specific needs of each setting. The purpose of this study was to develop a flexible, logistic triage model for children under five years of age that can be easily integrated into a digital platform and is operable with minimal clinical training. The digital triage tool can be used alongside ETAT to rapidly identify children at risk of developing severe infections, including sepsis upon arrival to the hospital. 

## Methods

This report was written in accordance with STROBE guidelines ^11^. A completed STROBE checklist is available ^12^.

### Ethics statement

This study was conducted at the Kenya Medical Research Institute (KEMRI)-Wellcome Trust Research Programme (KWTRP), and ethics approval was obtained by the KEMRI’s scientific and ethics review committee (certificate number, SERU/3407). The initial approval date was May 16 ^th^, 2017. Parents or caregivers of eligible children provided written informed consent prior to enrollment by the study nurse. Consent was deferred in emergency cases and taken after the child was stable to avoid introducing any delays. 

### Population

This prospective observational study was conducted between January and June 2018 at the pediatric outpatient department of Mbagathi County Referral hospital in Nairobi, Kenya. Mbagathi County Hospital is a first-referral level (district) hospital located in Nairobi in the neighbourhood of a high-density urban informal settlement. During a typical working shift, the outpatient area has nutritionists who take anthropometric measurements, a single nurse who conducts triage, plus providing treatments such as oral rehydration, and another nurse in the emergency area who administers emergency treatment, and one or two non-degree trained clinicians (clinical officers) who provide consultation, prescribe treatments, and make decisions on admissions. The outpatient department (OPD) serves over 20,000 children per year and admits approximately 2,500 pediatric patients per year. 

### Eligibility

Children aged 2–60 months seeking treatment for an acute illness at Mbagathi hospital on weekdays between 8:00 am and 5:00 pm were eligible for enrollment. Patients coming for elective procedures, such as elective surgery or for cardiac follow up, were excluded from the study. Patients presenting for elective care or treatment for chronic illnesses were excluded from the study.

### Study procedures

A study nurse competent to provide care and attend to emergencies was recruited and trained on study specific procedures and research ethics. The study nurse was expected to assist with emergency resuscitation if required but not expected to perform routine duties. Following introduction and orientation to hospital staff, the study nurse was stationed at the OPD, alongside hospital staff (nurses and clinicians). Children who presented to the OPD during study hours that did not require emergency treatment were screened for eligibility by the study nurse. For emergency cases (determined by hospital staff), treatment was started immediately, and data collection by the study nurse began only after emergency treatment initiation.

After consent and enrollment, the study nurse obtained patient history of presenting illness and performed clinical examination using a standardised checklist. The study nurse then entered all observations into a mobile data collection app on a tablet. This app recorded automated measurement of oxygen saturation and heart rate data using a pulse oximeter (LionsGate Technologies, Inc.) attached to the tablet and respiratory rate via the embedded RRate application ^13^. A total of 17 continuous variables and 37 categorical variables were selected for capture (54 in total), including patient demographics, anthropometric measurements, vitals, and clinical signs and symptoms ( Table 1). The patient was then reviewed by the hospital clinician on duty at the OPD who, without access to the study data, assessed the child, allocated treatment, and independently decided on whether or not to admit the patient or continue outpatient management. The study nurse recorded the clinician’s decision on the tablet. Study procedures did not delay care. 

**Table 1.** Candidate predictor variables (N=54). Transformed oxygen saturation = 4.314× log _10_(103.711–SpO _2_)–37.315 Abbreviation: AVPU: Alert, Voice, Pain, Unresponsive.

Patient Characteristics
Age in months
Male sex
Urgent referral status
Data collected after emergency treatment
Length of illness in days prior to admission
Anthropometrics
Weight in kg
Height in cm
Weight-for-height z-score
Weight-for-age z-score
Left mid-upper arm circumference in centimeters
Vitals
Axillary temperature in degrees Celsius
Pulse rate
Respiratory rate
Oxygen saturation (raw)
Oxygen saturation (transformed based on saturation gap*)
Respiratory distress
Chest indrawing
Apnoea
Central cyanosis
Difficulty breathing (parent reported)
Obstructed breathing
Nasal flaring
Grunting
Wheezing
Stridor
Acidotic breathing
Head nodding/bobbing
Cough
Cough duration in days prior to admission (parent reported)
**Circulation**
Capillary refill time ≥ 2 seconds
Weak, rapid pulse
Pallor (palmar, oral or conjunctival)
Skin warm at elbow or shoulder
Neurological
AVPU (patient does not respond to voice, pain or is unresponsive)
Difficulty feeding (parent reported)
Cannot drink or breastfeed
Irritability/restlessness
Convulsing now, actively
Convulsions (parent reported, history of convulsions)
Convulsion frequency in the past 24 hours (parent reported)
Newly onset hemiparesis
Gastrointestinal/Genitourinary
Diarrhoea (parent reported)
Diarrhoea duration in days prior to arrival (parent reported)
Vomiting (parent reported)
Vomiting frequency in the past 24 hours (parent reported)
Jaundice
Malnutrition
Visible severe wasting
Oedema of Kwashiorkor on feet, knees or face
Dehydration
Lateral abdominal skin pinch (delayed elasticity observed)
Sunken eyes
Infection
Fever (Axillary temperature > 38°C)
Fever duration in days prior to admission (parent reported)
Trauma
Uncontrolled bleeding
Other
Lethargy/reduced activity level (parent reported)
Appears in severe pain

For those children who were sent home, a telephone interview was conducted 14 days post-discharge to determine to determine 1) mortality status, 2) whether the child completely recovered from the illness, 3) if the child returned to a hospital or health center seeking help for the same illness, 4) if the child was admitted to a hospital or health center for the same illness.

### Outcome

The primary outcome was admission to hospital for greater than or equal to 24 hours. After assessing the child, the attending clinician independently decided on whether to admit the patient for further care in the hospital.

### Data management

Each participant was assigned a unique study identification number upon registration. Clinical observations and vital sign measurements were collected with a custom, password protected application, on a Dell Venue 7 ^®^ tablet and uploaded every day to a secure REDCap database ^14^, hosted on a KWTRP server.

### Statistical analysis

All statistical analyses were performed using R (3.5.1) ^15^.

### Candidate predictor variables

Candidate predictor variables were selected based on a combination of a literature review, availability, and ease of measurement in resource-limited facilities ^16^. A physiological transformation of the oxygen saturation (using a virtual shunt concept) was used to address the non-linear relation between oxygen saturation and impairment of gas exchange. Transformation was based on the saturation gap [49.314× log _10_(103.711– *SpO2*)–37.315], which has been demonstrated to improve the fit of logistic regression models ^17^. This transformed SpO _2_ was not available to the clinician but only calculated during analysis. Anthropometric z-scores were also calculated during analysis (computed via the zscorer package in R; v6.0-79, https://cran.r-project.org/web/packages/zscorer/zscorer.pdf). All variables were assessed using univariate logistic regression to estimate their level of association with the outcome.

### Missing data

Participants missing greater than 50% of predictors or missing the outcome variable were excluded from multivariate analysis. The remainder of missing data were assumed to be missing at random and imputed using multiple imputations by chained equations (MICE) ^18^. Ten imputed data sets were created and checked visually for similarity. Model development procedures were performed separately on each imputed data set and the results were pooled using Rubin’s Rules ^19^. If missingness was minimal, measures to evaluate model performance would be performed on one randomly selected imputed data set.

### Model development

Candidate predictors with less than 10 events per variable were not selected for inclusion in the final model to reduce the potential of overfitting ^20^. When similar information was collected both continuously and categorically, continuous variables were preferred ^20^. Continuous variables were assessed graphically for linear associations with the outcome and transformed where appropriate. Predictors to be included in the final model were selected using recursive feature elimination (RFE) ^21^ with repeated 10-fold cross validation (computed via the caret package in R; v6.0-79) ^22^. RFE eliminates features by fitting the model multiple times and at each step, removing the weakest predictors, determined by the coefficient attribute of the fitted model. The best subset of predictors is based on the model with the lowest cross validation error. Further inclusion into the list of variables was made based on clinical knowledge. The list of predictors included in the final model was checked for collinearity indicated as variance inflation factor > 5 or absolute correlation coefficient > 0.9. 

### Model discrimination

Model performance was primarily estimated as the area under the receiver operating curve (AUC). Low- and high-risk thresholds were selected to stratify participants into three triage groups (non-urgent, priority, emergency). The low-risk threshold was selected with the goal to maximize sensitivity in order to limit misclassification of emergency and priority cases as non-urgent (avoiding false-negatives). Specificity was used for selection of the high-risk threshold to maximize correct classification of emergency cases (avoiding false positives) in order to optimize resource utilization such that children in need of immediate treatment do not experience delays. A risk stratification table was used to evaluate model classification accuracy, defined as the ability of the model to separate the population into risk strata, such that cases with and without outcomes are more likely to be in the higher and lower risk strata, respectively. Performance characteristics (sensitivity, specificity, positive and negative predictive values, and positive and negative likelihood ratios) were calculated for each triage group. A five-time repeated 10-fold cross validation procedure was applied to all performance evaluation measures and results were pooled to provide a single estimate ^20^.

### Model calibration

Calibration was assessed with the GiViTI calibration belt and the associated likelihood based test ^23– 25^. The calibration belt is a graphical representation of the relationship between the estimated probabilities and observed outcome rates of a fitted polynomial logistic regression model.

## Results

### Participants

**Figure 1.**
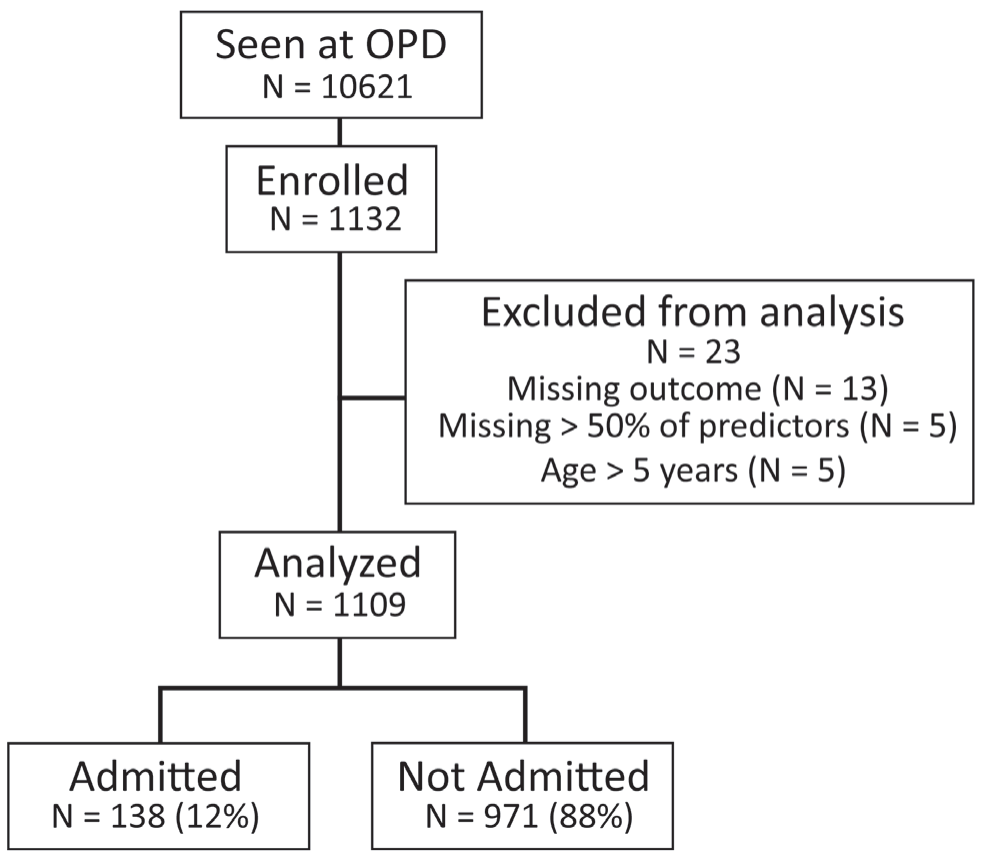
Flowchart of study population and distribution of outcomes. OPD, outpatient department.

Over the 6-month recruitment period, 10,621 children were seen at the OPD and a sample of 1,132 participants were enrolled in the study ( Figure 1). Of these, 23 were excluded from multivariable analysis as they were older than five years (N=5), missing more than half of the predictor variables (N=5) or missing the outcome (N=13). Demographic data, alongside all other variables measured, are available as *Underlying data* ^26^.

The median age of admitted participants (N=138) was 13 months (IQR 8 to 23), compared to a median age of 16 months (IQR 10 to 30) for discharged participants (N=971) ( Table 2). The proportion of males in the admitted and discharged groups was 62% and 45%, respectively. Rate of overnight hospital admission was 12% (N=138), and the most common reason for admission was pneumonia (N=84) ( Table 3). Of the admitted participants, 37% were urgent referral cases and 86% were consented after emergency treatment ( Table 2). Results from the 14 day follow up call revealed that 837 (86%) of discharged participants completely recovered from the illness and 89 (9%) returned to a hospital or health center for reassessment, of which 24 were admitted ( Table 4). Mortality outcomes for both the admitted participants (N=4) and discharged participants (N=2) were minimal ( Table 4).

**Table 2.** Predictor variable distribution for admitted and non-admitted children. *Values include medians (quartiles) for continuous predictors and number of children (percentage) for dichotomous predictors. OR, odds ratio; AUC, area under the curve.

Predictor Variables	Missing	Admission Required *	Admission not required *	OR (95% CI)	AUC (95% CI)	p Value
Continuous (N = 17)						
Age (months)	0	13 (8-23)	17 (2-30)	0.97 (0.96 to 0.99)	0.61 (0.56 to 0.66)	<0.001
Length of illness (days)	4	3 (2-7)	4 (2-7)	1.00 (0.96 to 1.03)	0.52 (0.47 to 0.57)	0.827
Weight (kg)	6	8 (6-10)	10 (8-12)	0.21 (0.03 to. 6.00)	0.69 (0.62 to 0.73)	<0.001
Height (cm)	14	71 (65-80)	77 (70-88)	0.04 (0.01 to 4.92)	0.64 (0.59 to 0.69)	<0.001
Left mid-upper arm circumference (cm)	17	12 (12-14)	14 (13-15)	0.64 (0.57 to 0.71)	0.68 (0.64 to 0.73)	<0.001
Axillary temperature (°C)	8	37 (36-38)	36 (36-37)	2.24 (1.87 to 2.69)	0.71 (0.65 to 0.76)	<0.001
Oxygen saturation (raw)	35	93 (87-97)	95 (92-97)	0.95 (0.92 to 0.97)	0.58 (0.52 to 0.64)	<0.001
Oxygen saturation (transformed)	35	13 (3-23)	9 (3-15)	1.03 (1.01 to 1.05)	0.58 (0.52 to 0.64)	<0.001
Respiratory rate (breaths per minute)	6	54 (40-67)	43 (36-54)	1.04 (1.03 to 1.05)	0.65 (0.60 to 0.70)	<0.001
Pulse rate (beats per minute)	35	155 (136-175)	140 (125-156)	1.02 (1.02 to 1.03)	0.64 (0.59 to 0.70)	<0.001
Weight-for-age z-score	11	-1 (-2-0)	-1 (-2-0)	0.93 (0.84 to 1.04)	0.46 (0.41 to 0.51)	0.200
Weight-for-height z-score	18	0 (-2-0)	0 (-1-0)	0.67 (0.58 to 0.77)	0.41 (0.36 to 0.47)	<0.001
Fever duration (days)	1	3 (2-5)	3 (1-5)	1.13 (1.08 to 1.18)	0.69(0.65 to 0.73)	<0.001
Vomiting frequency (per 24 hours)	1	3 (2-4)	3 (2-3)	1.09 (1.00 to 1.20)	0.52 (0.48 to 0.58)	0.053
Diarrhoea duration (days)	0	2 (2-5)	3 (2-5)	1.05 (0.99 to 1.20)	0.55 (0.51 to 0.60)	0.078
Convulsion frequency (per 24 hours)	4	2 (2-3)	1 (1-3)	1.83 (1.64 to 2.08)	0.57 (0.54 to 0.60)	<0.001
Cough duration (days)	1	3 (2-5)	4 (2-7)	0.97 (0.99 to 1.12)	0.48 (0.43 to 0.53)	0.191
Dichotomous (N = 37)						
Urgent referral	3	51 (37)	23 (2)	5.30 (3.58 to 7.81)	0.65 (0.56 to 0.74)	<0.001
Data collected after emergency treatment	7	120 (86)	265 (23)	17.06 (10.55 to 29.09)	0.80 (0.74 to 0.85)	<0.001
Fever	2	99 (71)	344 (35)	4.53 (3.09 to 6.76)	0.67 (0.60 to 0.75)	<0.001
Vomiting	3	52 (37)	338 (34)	1.14 (0.79 to 1.65)	0.51 (0.44 to 0.59)	0.473
Diarrhoea	2	52 (37)	261 (27)	1.67 (1.15 to 2.42)	0.56 (0.48 to 0.63)	<0.01
Convulsions	6	25 (18)	30 (3)	6.61 (3.71 to 11.68)	0.57 (0.48 to 0.67)	<0.001
Convulsing now	10	3 (2)	8 (1)	3.60 (0.95 to 11.58)	0.51 (0.42 to 0.60)	<0.050
Cough	3	92 (66)	646 (66)	1.00 (0.69 to 1.47)	0.44 (0.38 to 0.51)	0.999
Lethargy	10	121 (87)	452 (46)	8.81 (5.31 to 15.62)	0.70 (0.65 to 0.75)	<0.001
Indrawing	10	54 (39)	19 (2)	32.24 (18.58 to 58.21)	0.69 (0.59 to 0.78)	<0.001
Difficulty breathing	2	82 (59)	240 (24)	4.51 (3.13 to 6.56)	0.67 (0.60 to 0.75)	<0.001
Irritability or restlessness	12	64 (46)	172 (18)	4.05 (2.79 to 11.58)	0.64 (0.56 to 0.73)	<0.001
Cannot drink or breastfeed	10	49 (35)	81 (8)	0.15 (0.11 to 0.24)	0.63 (0.55 to 0.73)	<0.001
Nasal flaring	9	40 (29)	24 (2.4)	16.12 (9.40 to 28.21)	0.63 (0.54 to 0.73)	<0.001
Pallor	12	52 (37)	133 (14)	3.85 (2.60 to 5.67)	0.62 (0.53 to 0.71)	<0.001
Appears in severe pain	10	40 (29)	58 (6)	6.43 (4.07 to 10.11)	0.61 (0.52 to 0.70)	<0.001
Lateral abdominal skin pinch	10	30 (22)	53 (5)	4.81 (2.93 to 7.82)	0.58 (0.49 to 0.67)	<0.001
Weak, rapid pulse	12	27 (19)	37 (4)	0.16 (0.10 to 0.27)	0.58 (0.49 to 0.67)	<0.001
Sunken eyes	11	42 (30)	163 (17)	2.17 (1.45 to 3.22)	0.57 (0.48 to 0.65)	<0.001
Acidotic breathing	10	21 (15)	24 (2)	6.80 (3.66 to 12.52)	0.56 (0.47 to 0.66)	<0.001
Skin warm	10	20 (14)	25 (3)	6.17 (3.30 to 11.36)	0.56 (0.47 to 0.65)	<0.001
Alert, Verbal, Pain, Unresponsive scale	12	16 (12)	17 (2)	7.37 (3.60 to 15.02)	0.55 (0.46 to 0.64)	<0.001
Difficulty feeding	2	78 (56)	476 (49)	1.37 (0.96 to 1.96)	0.54 (0.48 to 0.60)	0.089
Grunting	10	26 (19)	106 (11)	1.90 (1.16 to 3.00)	0.54 (0.45 to 0.63)	<0.010
Visible severe wasting	10	14 (10)	23 (2)	4.66 (2.28 to 9.19)	0.54 (0.45 to 0.63)	<0.001
Wheezing	9	26 (19)	249 (25)	0.68 (9.40 to 28.21)	0.53 (0.47 to 0.60)	0.090
Male Sex	4	85 (62)	440 (45)	1.27 (0.88 to 1.82)	0.53 (0.47 to 0.59)	0.206
Head nodding/bobbing	10	8 (6)	13 (1)	4.54 (1.77 to 10.98)	0.52 (0.43 to 0.61)	<0.001
Oedema	0	7 (5)	15 (2)	14.28 (1.47 to 11.97)	0.52 (0.43 to 0.61)	<0.010
Central cyanosis	9	4 (3)	13 (1)	2.20 (0.61 to 6.33)	0.51 (0.42 to 0.60)	0.173
Jaundice	10	4 (3)	15 (2)	1.90 (0.54 to 5.34)	0.51 (0.42 to 0.60)	0.259
Newly onset hemiparesis	11	1 (1)	6 (1)	2.37 (0.34 to 10.39)	0.50 (0.41 to 0.59)	0.294
Obstructed Breathing	9	6 (4)	38 (4)	1.11 (0.41 to 2.51)	0.48 (0.40 to 0.57)	0.805
Stridor	8	9 (6)	59 (6)	1.08 (0.49 to 2.12)	0.48 (0.40 to 0.56)	0.836
Capillary refill time (≥ 2 seconds)	11	27 (19)	31 (3)	7.38 (4.23 to 12.83)	0.58 (0.48 to 0.68)	<0.001
Apnoea	9	0 (0.0)	3 (0)	low event rate (N=3)		0.980
Uncontrolled bleeding	11	0 (0.0)	3 (0)	low event rate (N=3)		0.980

**Table 3.** Profile of reasons for admission.

Admission Reason	Frequency (%)
Pneumonia	84 (61)
Malnutrition	28 (20)
Dehydration	22 (16)
Diarrhea	16 (12)
Malaria	10 (7)
Convulsions/convulsive disorder	10 (7)
Anemia	6 (4)
Sickle Cell Disease	6 (4)
Meningitis	3 (2)
Ricketts	3 (2)
Asthma	2 (1)
Trauma	1 (1)
Aspiration	1 (1)
Bronchiolitis	1 (1)
Hypoglycemia	1 (1)
Neonatal Sepsis	1 (1)
Obstructive Jaundice	1 (1)
RTI	1 (1)
Shock	1 (1)
Vaso-occlusive crisis	1 (1)
Vomiting	1 (1)

**Table 4.** General characteristics of admitted and discharged participants.

Admitted Participants (N=138)	Frequency (%)	Discharged Participants (N=971)	Frequency (%)
**Age (months)**		**Age** **(months)**	
<12	64 (46.4)	<12	306 (31.5)
12–24	45 (32.6)	12–24	318 (32.7)
24–36	13 (9.4)	24–36	158 (16.3)
36–48	5 (3.6)	36–48	109 (11.2)
48–60	9 (6.5)	48–60	74 (7.6)
60	2 (1.4)	60	6 (0.6)
**Male Sex**	85 (61.6)	**Male Sex**	440 (45.3)
**Mortality**	4 (2.9)	**Mortality**	2 (0.2)
**Length of** **hospital stay** **(days)**	****	**Recovered**	837 (86.2)
<3	11 (8.0)	**Returned to** **hospital**	89 (9.2)
3–5	43 (31.2)	**Readmitted**	24 (2.5)
6–10	64 (46.4)		
>10	20 (14.5)		

Three categorical variables, apnoea, bleeding, and newly onset hemiparesis, had events per variable below 10 and were not included in multivariable analysis ( Table 2). Missing observations were minimal (≤ 3% missing per predictor) and results were near identical across each of the 10 imputed data sets ( Table 2). Univariate analysis revealed that many variables had a significant association with the outcome ( Table 2). Of these variables, data collected after emergency treatment had the highest AUC: 0.80 (95% CI 0.74 to 0.85).

### Final multivariable model

**Figure 2.**
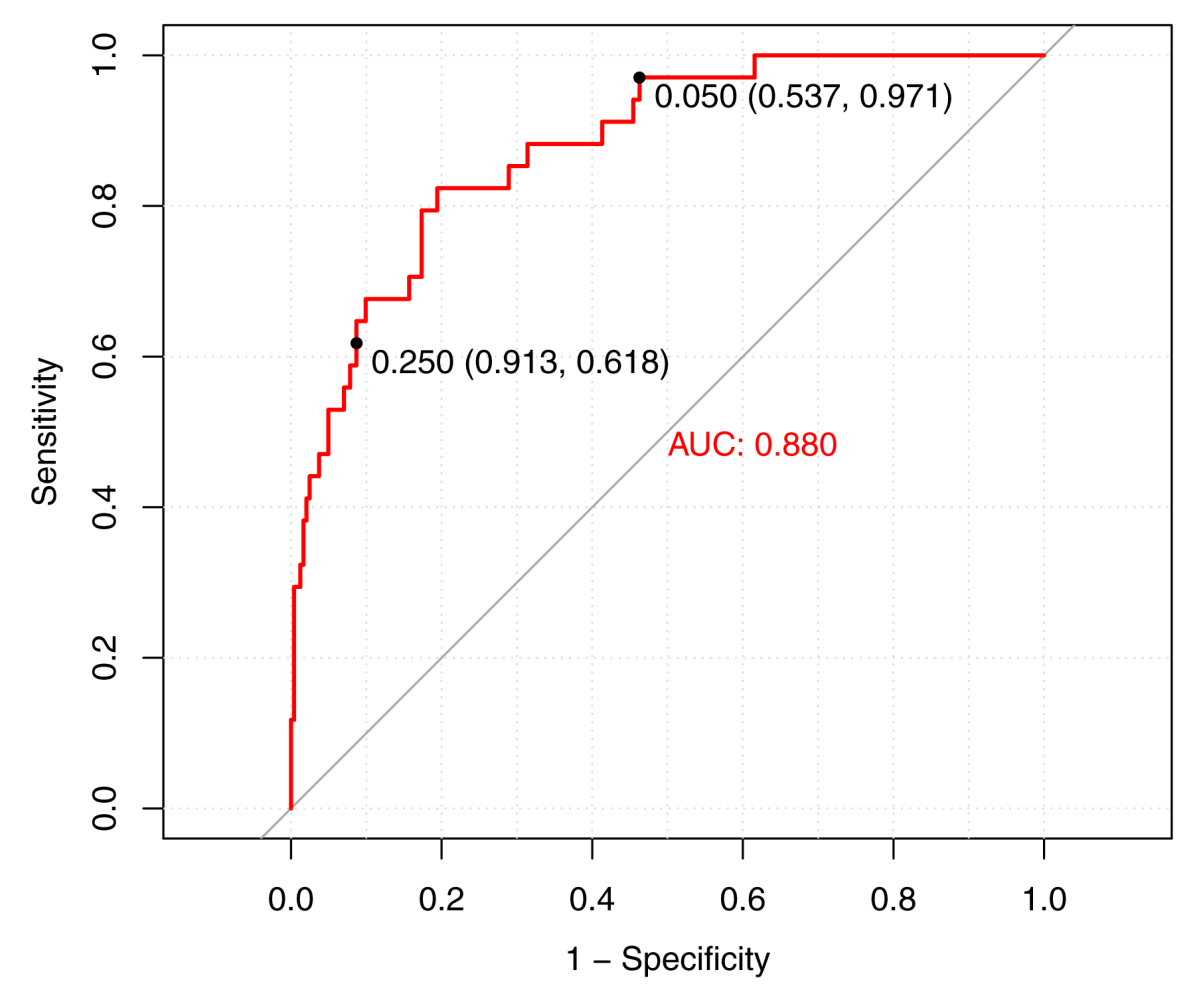
Cross validated receiver operating characteristic curve of the final model in the study cohort. Points represent low risk (0.05) and high risk (0.25) thresholds. AUC, area under the curve.

The final model was reduced to 8 predictor variables ( Table 5) and achieved an AUC of 0.88 (95% CI 0.82 to 0.94) ( Figure 2). The final model equation was: logit (p)= -3.45 +(-0.006,weight)+(1.51,lethargy)+(-0.03,MUAC)+(1.19,cannot drink/breastfeed)+(-0.004,transformed oxygen saturation)+(0.05,temperature)+(1.15,difficulty breathing)+(0.006,pulse rate).

**Table 5.** Odds ratios of predictors in the final model. * *p* < 0.05, ** *p* < 0.0001. Transformed oxygen saturation = 4.314×log _10_ (103.711–SpO _2_)–37.315. OR, odds ratio; CI, confidence interval; MUAC, mid-upper arm circumference.

Predictors	Regression estimate	OR(95% CI)
(Intercept)	-3.447	
Weight (kg)	-0.006 **	0.994 (0.991 to 0.998)
Lethargy	1.512 **	4.537 (3.784 to 5.470)
MUAC (cm)	-0.027 **	0.973 (0.967 to 0.979)
Cannot drink or breastfeed	1.188 **	3.280 (2.807 to 3.833)
Transformed oxygen saturation	-0.004 *	0.997 (0.987 to 1.000)
Axillary temperature (°C)	0.046 **	1.047 (1.039 to 1.054)
Difficulty breathing	1.149 **	3.516 (2.755 to 3.62)
Pulse rate (bpm)	0.006 **	1.006 (1.003 to 1.009)

The model, at a low risk threshold of 5%, had a sensitivity of 97% (95% CI 91% to 99%), and a specificity of 54% (95% CI 45% to 58%) ( Table 6) ( Figure 2). In the model derivation cohort, the positive predictive value was 22% (95% CI 20% to 26%), and the negative predictive value was 99% (95% CI 98% to 100%). At a high-risk threshold of 25% the model attained a specificity of 91% (95% CI 83% to 95%) and a sensitivity of 62% (95% CI 53% to 79%). The positive and negative predictive values were 50% (95% CI 39% to 64%) and 94% (95% CI 93% to 97%), respectively.

**Table 6.** Risk stratification table using three triage groups. Computed using the upper limit, median and lower limit of the risk threshold range for the non-urgent, priority and emergency categories respectively.

Triage Category:	Non-Urgent	Priority	Emergency
Risk threshold range	≤ 5	5 < r < 25	≥ 25
Participants, n (%)	519 (47)	418 (38)	172 (16)
Participants with outcome, n (%)	11 (8)	48 (35)	79 (57)
TP:FP	1:19	3:17	1:3
Sensitivity (95% CI)	0.97 (0.91 to 0.99)	0.76 (0.61 to 0.88)	0.62 (0.53 to 0.79)
Specificity (95% CI)	0.54 (0.45 to 0.58)	0.79 (0.74 to 0.84)	0.91 (0.83 to 0.95)
NPV (95% CI)	0.99 (0.98 to 1.00)	0.97 (0.94 to 0.99)	0.94 (0.93 to 0.97)
PPV (95% CI)	0.22 (0.20 to 0.26)	0.37 (0.30 to 0.45)	0.50 (0.39 to 0.64)
NLR (95% CI)	0.05 (0.01 to 0.38)	0.26 (0.13 to 0.50)	0.42 (0.27 to 0.64)
PLR (95% CI)	2.10 (1.82 to 2.44)	4.16 (3.04 to 5.68)	6.88 (4.23 to 11.20)

**Figure 3.**
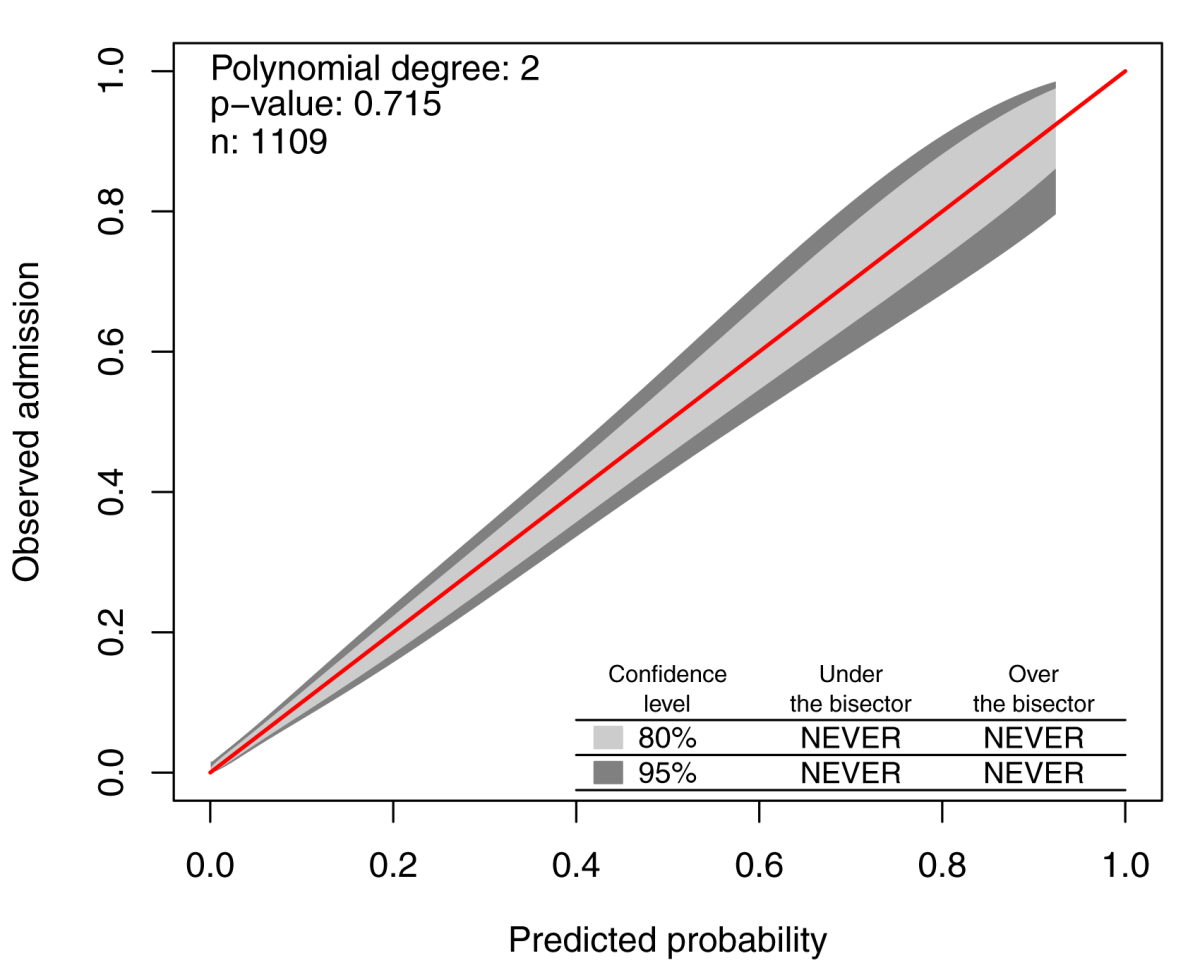
Calibration belt of the final model. The 45-degree bisector represents the identity between predicted probabilities and observed responses. The 80% and 95% confidence level calibration belt are plotted, in light and dark grey respectively. The test’s p-value, the sample size *n,* and the polynomial order *m* of the calibration curve are reported in the top left corner.

The calibration belt and associated likelihood ratio-based test suggest the model is well calibrated (p = 0.715) ( Figure 3). The majority of participants identified in the non-urgent category (46.7%, *n* = 519) had low rates of admission (7.9%, *n* = 11) ( Table 6). Participants in the emergency category (15.5%, *n* = 172) had high admission rates (57.2%, *n* = 79). This is much greater than the population prevalence of admission (12.4%), as reflected by the high positive likelihood ratio (PLR) associated with this category (6.88, 95% CI 4.23 to 11.20).

## Discussion

### Key results

We have developed, and internally validated, a prediction model for triage of children under-five years of age presenting to an outpatient department based on the need for hospital admission. The final model includes eight predictor variables, five of which are objectively measurable (transformed oxygen saturation, pulse rate, temperature, weight, MUAC), and three of which are parent reported (lethargy, inability to drink/breastfeed, difficulty breathing). This simple model is derived from predictors that are readily available globally at relatively low cost and can be easily measured by low skilled frontline health workers. The predictors included in the model reflect what has been observed in previous studies ^27– 29^ and what is often included in international guidelines ^30^. After internal validation, the model affords high discrimination, with an AUC of 0.88 (95% CI 0.82 to 0.94) and good calibration (p = 0.715).

### Clinical interpretation

Triage of children is typically categorized into three levels of risk (emergency, priority, and non-urgent). If using a risk threshold of 5% to differentiate non-urgent from priority and emergency cases, the model showed 97% sensitivity and 54% specificity ( Figure 2). The high sensitivity demonstrates good ability of the model to accurately identify non-urgent cases (rule out). This is not without a cost of specificity, evident in the ratio of one true positive to 19 false positives ( Table 6). However, in the case of triage this trade off may be acceptable to ensure that priority and emergency cases are not misclassified and treated as non-urgent. This is reflected by the negative likelihood ratio which suggests that the 47% of participants that were categorized as non-urgent are 20 times less likely be in need of hospital admission compared to participants categorized as priority or emergency ( Table 4). 

Using a risk threshold of 25% to identify emergency cases, the model attained 91% specificity and 62% sensitivity ( Figure 2). A highly specific model can be useful in correctly ruling in participants categorized as emergency, illustrated by a ratio of one true positive to only three false negatives ( Table 6). In the case of identifying emergency cases, high specificity is crucial to optimize time and resource allocation to children, who are truly in need of emergency treatment. The positive likelihood ratio suggests that the 16% of participants in the emergency category are 6.88 times more likely to need hospital admission compared to participants categorized as priority ( Table 6). The associated sensitivity cost is less important in this case as correct identification of emergency cases holds precedence and children who are incorrectly classified as priority cases will still receive prompt assessment.

Of children who required hospital admission, 92% were assigned into the priority and emergency triage categories, while the majority of non-outcome cases were assigned into the non-urgent category ( Table 6). This suggests good risk stratification capability.

### Strengths and limitations

This study represents a step forward in strengthening triage systems in LMICs by presenting a data-driven prediction model to be integrated into a real time electronic digital platform. There is increasing evidence to suggest that mHealth (use of mobile devices with software applications to provide health services and manage patient information) can be used to strengthen health systems ^31^. The computing power and display capability of even the entry level smartphones in low resource settings can be used as platforms to implement clinical prediction models ^32^. The digital platform also allows for real time monitoring of user performance and compliance and optimization of work flow. In addition, pulse oximetry can be conducted with mobile device by attaching low-cost sensors to enable objective measurements and alleviate the need to perform manual data entry of values read from a separate monitor ^29, 32, 33^. The inclusion of RRate, an app for measure respiratory rate by tapping on the screen also enables faster collection of respiratory rate with less effort than counting breaths ^11^. The data-driven model is comprised of eight objectively measurable or parent reported variables, minimizing need for subjective assessment and clinical expertise. Integration of this eight-predictor model into a mobile device could result in a simple, low cost triage tool that is easily implementable in low resource health facilities. 

The objectivity of five of the predictors would significantly support their adoption by lower skilled health workers. Having one study nurse perform data collection prevented introduction of inter-examiner measurement bias. However, due to time constraints, the study nurse did not have time to record information on participants that did not meet the inclusion criteria. The outcome variable (decision to admit) which was based on the opinion of the facility clinician on duty, was subject to inter-examiner variability. Opinions between physicians vary and are impacted by training, resource constraints, exposure and expertise.

A significant limitation of this study was the use of admission as a surrogate for acuity. Need for hospital admission is difficult to assess and may not accurately reflect a state of critical illness in children. We accounted for this by defining a positive outcome as admission for at least 24 hours to filter out those non-critically ill cases. We also conducted a 14-day post-discharge follow up call to identify children inappropriately sent home and found that both mortality and readmission rates were minimal ( Table 4). 

Furthermore, many predictors used in modelling were likely used by the facility physicians in outcome ascertainment. This inherently biases the model in favour of the chosen variables. Future studies should capture hospital outcomes as well to help inform the triage model.

Some risk factors could not be used in multivariable analysis due to low prevalence in the study population. This may indicate need for a study with a larger sample size. When a single sign or symptom with low population prevalence, such as unconsciousness, is well known to indicate risk this should be used as a danger sign prior to the use of any risk prediction tool. Risk prediction within a mobile app is only necessary when multiple predictors are required to augment the prediction. Alternatively, if these danger signs are easy to assess and strongly correlated with admission, these predictors may be treated as independent triggers for admission. This cascade of decision rules can be readily implemented in a digital platform with the complexity hidden from the user.

A further limitation was the poor signal quality for oxygen saturation (50% of participants had a signal quality index of less than 80%). Nevertheless, the finding of oxygen saturation as a strong predictor of overnight hospital admission is consistent with existing literature ^27, 34^. This could be improved with enhanced training and optimized technology.

Finally, the lack of external validity poses a significant limitation to this study. The model is currently being validated in an independent multi-site study that will include clinical implementation to assess performance in varied geographical locations, seasons, and with different disease prevalence and severity ^35^. 

## Conclusion

We developed a logistic triage model for rapid identification of critical illness in children at first contact. The triage model, comprised of five objectively measurable variables (transformed oxygen saturation, pulse rate, temperature, weight, MUAC) and three parent reported variables (lethargy, inability to drink/breastfeed, difficulty breathing) had good discrimination, calibration, and internal validation. The model can be easily integrated into a digital health platform and used with minimal clinical training. External validation is required prior to adoption. 

## Data availability

### Underlying data

Figshare: Derivation and internal validation of a data-driven prediction model to guide frontline health workers in triaging children under-five in Nairobi, Kenya. https://doi.org/10.6084/m9.figshare.9104918 ^26^.

### Reporting guidelines

Figshare: STROBE checklist for article ‘Derivation and internal validation of a data-driven prediction model to guide frontline health workers in triaging children under-five in Nairobi, Kenya’. 10.6084/m9.figshare.9037406 ^12^.

Data are available under the terms of the Creative Commons Attribution 4.0 International license (CC-BY 4.0).
