# Supplementary material for: Derivation and internal validation of a data-driven prediction model to guide frontline health workers in triaging children under-five in Nairobi, Kenya
Source: Wellcome Open Res. 2021 Apr 19;4:121. Originally published 2019 Aug 14. [Version 3] doi: 10.12688/wellcomeopenres.15387.3 (PMC8097734; doi:10.12688/wellcomeopenres.15387.3)
Supplement: Peer review history

# Peer review: Reviewer response for version 3

**DOI:** 10.21956/wellcomeopenres.18511.r43523

**Published:** 2021-05-04

**Authors:** Babar Hasan

**Affiliations:** 1 Department of Paediatrics and Child Health, Aga Khan University, Karachi, Pakistan

I am satisfied with the responses by the authors and the changes made. I also want to thank the authors for answering my queries very coherently and scientifically.

Is the work clearly and accurately presented and does it cite the current literature?

Yes

If applicable, is the statistical analysis and its interpretation appropriate?

Yes

Are all the source data underlying the results available to ensure full reproducibility?

Yes

Is the study design appropriate and is the work technically sound?

Yes

Are the conclusions drawn adequately supported by the results?

Partly

Are sufficient details of methods and analysis provided to allow replication by others?

Yes

Reviewer Expertise:

Pediatric cardiology, Quality improvement sciences

I confirm that I have read this submission and believe that I have an appropriate level of expertise to confirm that it is of an acceptable scientific standard.

# Peer review: Reviewer response for version 3

**DOI:** 10.21956/wellcomeopenres.18511.r43522

**Published:** 2021-04-29

**Authors:** Andrew Argent

**Affiliations:** 1 Department of Paediatrics and Child Health, University of Cape Town, Cape Town, South Africa

Thank you for the changes. I have no further suggestions for this paper.

Is the work clearly and accurately presented and does it cite the current literature?

Yes

If applicable, is the statistical analysis and its interpretation appropriate?

Yes

Are all the source data underlying the results available to ensure full reproducibility?

No

Is the study design appropriate and is the work technically sound?

Partly

Are the conclusions drawn adequately supported by the results?

Partly

Are sufficient details of methods and analysis provided to allow replication by others?

No

Reviewer Expertise:

I have experience in the evaluation of critically ill children for admission, particularly in areas of lower resource availability

I confirm that I have read this submission and believe that I have an appropriate level of expertise to confirm that it is of an acceptable scientific standard.

# Peer review: Reviewer response for version 2

**DOI:** 10.21956/wellcomeopenres.18080.r42880

**Published:** 2021-03-15

**Authors:** Babar Hasan

**Affiliations:** 1 Department of Paediatrics and Child Health, Aga Khan University, Karachi, Pakistan

It is well conducted work and does come up with a tool that has the potential of impact due to the simplicity of its use, device based data acquisition and simplicity of predictor variables with high accuracy.

 Following concerns can be addressed mainly by either changing some of the text in intro, discussion and conclusion or adding more details to the limitation section. This work is more at a proof of concept stage and needs larger validation studies and a possible RCT to truly reveal its worth.

 General comments:

- Keeping the eventual physician recommendation to admit as a surrogate to the gold standard which is actual mortality and morbidity may lead to several biases.
- A lot of their criticism of WHO-ETAT is that it is expert opinion based and the prediction power of each individual variable is not known. Additionally they mention that it is not real time and thus doesn’t have the ability to look at compliance and QC. Also they question generalizability of the tool as it is based on decision rules. Now if one was to look at their tool, it also has the exact same issues as the WHO-ETAT, how are they claiming that it is innovative or different. It was not developed as a real time tool. The tool had questions pretty similar to the WHO-ETAT. They haven’t demonstrated a tool which tracks compliance or quality. Additionally have they truly proven it to be better than WHO-ETAT for which an RCT is required?
- A little more description about the Mbaghati hospital where the study was conducted is needed. I want to know what is the spectrum of pediatric patients they deal with as inpatients, do they have ICU facilities, do they has pediatric surgical services etc? I need this information to judge the level of pediatric expertise in this hospital. This may determine the generalizability of their findings. Skills of a triage nurse in a well “oiled” pediatric hospital may be far different than someone in a remote primary health care facility. The tool may perform very differently in that setting.
- Were the anthropometric z-score derived using online calculators or was it built into the data capturing software thus automating its calculations.
- Fever of >38 degree was determined by the study nurse or as per parents report?
- The RRATE application which ingested data from the pulse oximeter, is it a free application? Does it work with any operating system? Is it compatible with other pulse ox devices?
- If primary outcome was 24hrs admission, how were patients who were sent home and died accounted for in the analysis. I mean these patients were the false negative of the screening tool.
- Did they do the 10 fold cross validation at both the feature selection and model prediction stages on the whole dataset or did they do a 70/30 data split to test their final model of an unseen 30% of the data?
- If the missing data was minimal (<3% missing per predictor), why did the authors do an imputation exercise?
- “Of these variables, data collected after emergency treatment had the highest AUC: 0.80 (95% CI 0.74 to 0.85).“ Isn’t this pretty intuitive. I mean whoever received emergent treatment (and thus they got their data collected after those emergent services were given) will get admitted. Thus, in my view, the clinical application of such a predictor variable is meaningless.
- Table 4, mortality in discharged patients was 2 while the parenthesis says (0%). It is 0.2%. I think one has to be accurate with such percentages especially if it represents a grave, rare event.
- “to suggest that mHealth (use of mobile devices with software applications to provide health services and manage patient information) can be used to strengthen health systems31. The computing power and display capability of even the entry level smartphones in low resource settings can be used as platforms to implement clinical prediction models32. The digital platform also allows for real time monitoring of user performance and compliance and optimization of work flow.” This is something the authors mention in their introduction and even as a conclusion. Their current data is not enough to drive the conclusion around the utility of this model as an mobile app. Additionally they can’t claim superiority over WHO- ETAT, for that they have to really do a proper trial.
-  “In addition, pulse oximetry can be conducted with mobile device by attaching low-cost sensors to enable objective measurements and alleviate the need to perform manual data entry of values read from a separate monitor”. It is true but sometimes that cross talk between the device output and auto-populating data into another software may not be that easy of device company agnostic. The commercial value of such a software which facilitates this cross talk may not be affordable.
- “The inclusion of RRate, an app for measure respiratory rate by tapping on the screen also enables faster collection of respiratory rate with less effort than counting breaths”. Again company proprietorship and device agnostic cross talk may preclude its generalizability.
- The greatest flaw in the model is that it excluded a very “serious” sign and symptoms like apnea, bleeding and new onset hemiparesis due to unavailability of data.

Is the work clearly and accurately presented and does it cite the current literature?

Yes

If applicable, is the statistical analysis and its interpretation appropriate?

Yes

Are all the source data underlying the results available to ensure full reproducibility?

Yes

Is the study design appropriate and is the work technically sound?

Yes

Are the conclusions drawn adequately supported by the results?

Partly

Are sufficient details of methods and analysis provided to allow replication by others?

Yes

Reviewer Expertise:

Pediatric cardiology, Quality improvement sciences

I confirm that I have read this submission and believe that I have an appropriate level of expertise to confirm that it is of an acceptable scientific standard, however I have significant reservations, as outlined above.

# Response

**Published:** 2021-03-31

**Authors:** Alishah M

Thank you for your comments!

 We have modified the introduction, discussion, and conclusion specifically to convey the message that we are not claiming superiority of this model over ETAT, but instead that we developed a model that has the potential to strengthen (not replace) existing triage systems. We acknowledge that external validation is needed before drawing any conclusions regarding model utility. In a study, currently in progress, we are conducting a clinical evaluation of this model:

 Mawji A, Li E, Komugisha C, *et al.*: **Smart triage: triage and management of sepsis in children using the point-of-care Pediatric Rapid Sepsis Trigger (PRST) tool.** *BMC Health Serv Res. *2020; **20**(1): 493.

**General comments:**

  

- **Keeping the eventual physician recommendation to admit as a surrogate to the gold standard which is actual mortality and morbidity may lead to several biases.**

We do agree with you. As the study was only funded for a duration of 6 months, we could not capture enough mortality outcomes (N=6 deaths) to conduct meaningful analysis of mortality as an outcome. In these cases, admission is often used as the surrogate. We have acknowledged the limitations associated with this in the manuscript:

*A significant limitation of this study was the use of admission as a surrogate for acuity. Need for hospital admission is difficult to assess and may not accurately reflect a state of critical illness in children (Strengths and limitations, Paragraph 3).*

  

- **A lot of their criticism of WHO-ETAT is that it is expert opinion based and the prediction power of each individual variable is not known. Additionally, they mention that it is not real time and thus doesn’t have the ability to look at compliance and QC. Also, they question generalizability of the tool as it is based on decision rules. Now if one was to look at their tool, it also has the exact same issues as the WHO-ETAT, how are they claiming that it is innovative or different. It was not developed as a real time tool. The tool had questions pretty similar to the WHO-ETAT. They haven’t demonstrated a tool which tracks compliance or quality. Additionally, have they truly proven it to be better than WHO-ETAT for which an RCT is required?**

We agree with you. We do not wish to claim that the tool is superior to ETAT or should be used as a replacement to ETAT. We are suggesting that digital triage tools can be used in conjunction with existing care pathways to strengthen existing systems. The introduction has been updated to reflect this:

*One solution to these shortcomings is the use of a digital, data-driven approach to strengthen triage systems at first contact. Digital health platforms can facilitate quality improvement, while data-driven algorithms are easily updatable with emergence of new information and can be optimized to meet the specific needs of each setting. The purpose of this study was to develop a flexible, logistic triage model for children under five years of age that can be easily integrated into a digital platform and is operable with minimal clinical training. The digital triage tool can be used alongside ETAT to rapidly identify children at risk of developing severe infections, including sepsis upon arrival to the hospital (Introduction, Paragraph 4).*


** **

- **A little more description about the Mbaghati hospital where the study was conducted is needed. I want to know what is the spectrum of pediatric patients they deal with as inpatients, do they have ICU facilities, do they has pediatric surgical services etc.? I need this information to judge the level of pediatric expertise in this hospital. This may determine the generalizability of their findings. Skills of a triage nurse in a well “oiled” pediatric hospital may be far different than someone in a remote primary health care facility. The tool may perform very differently in that setting.**

We have updated the manuscript to include more about Mbagathi hospital:

*This prospective observational study was conducted between January and June 2018 at the pediatric outpatient department of Mbagathi County Referral hospital in Nairobi, Kenya. Mbagathi County Hospital is a first-referral level (district) hospital located in Nairobi in the neighbourhood of a high-density urban informal settlement. During a typical working shift, the outpatient area has nutritionists who take anthropometric measurements, a single nurse who conducts triage, plus providing treatments such as oral rehydration, and another nurse in the emergency area who administers emergency treatment, and one or two non-degree trained clinicians (clinical officers) who provide consultation, prescribe treatments, and make decisions on admissions. The outpatient department (OPD) serves over 20,000 children per year and admits approximately 2,500 pediatric patients per year (Methods, Population).*

 A more extensive description is below:

 Mbagathi County Hospital is a first-referral level (district) hospital located in Nairobi in the neighbourhood of a high-density urban informal settlement. The hospital has outpatient services that attends to patients coming from home seeking medical care or referred from lower-level facilities that provide only outpatient services. Patients arriving at the outpatient department who need emergency care are taken straight to the emergency/resuscitation room for emergency treatment.  The outpatient area also serves patients coming for clinical follow-ups. During a typical working shift, the outpatient area has nutritionists who take anthropometric measurements, a single nurse who conducts triage, plus providing treatments such as oral rehydration, and another nurse in the emergency area who administers emergency treatment, and 1-2 non-degree trained clinicians (clinical officers) who provide consultation, prescribe treatments, and make decisions on admissions.  The hospital has a newborn ward, admitting sick newborns born within the hospital aged less 1 month, and a paediatric ward that admits other children aged up to 12 years.  The paediatric ward is a general ward with one room closest to the nursing station equipped with portable oxygen to attend to acutely ill who may need oxygen or closer observation.  Like many hospitals, the hospital has shortage of skilled health workforce and it is usual to have about 2 nurses attending to even up to 40 patients and covering all nursing tasks.  The clinical team in the paediatric ward is led by one paediatrician who works with intern clinicians (doctors and clinical officers). The hospital does not have a high dependency unit (HDI) or intensive care unit (ICU) and patients requiring these levels of care are referred.

  

- **Were the anthropometric z-score derived using online calculators or was it built into the data capturing software thus automating its calculations.**
**  **

Anthropometric z-scores were calculated using the zscorer package in R as it was unnecessary to calculate them at the time of data collection. When implemented as a digital platform this would be built in and automated. Our team has already included this in other digital tools. The manuscript has been updated to include this information:

*Anthropometric z-scores were also calculated during analysis (computed via the zscorer package in R; v6.0-79,* *https://cran.r-project.org/web/packages/zscorer/zscorer.pdf* *) (Methods, Candidate predictor variables)*

  

- **Fever of >38 degree was determined by the study nurse or as per parent’s report?**

This was an observed variable. Axillary temperature was measured by the study nurse.

  

- **The RRATE application which ingested data from the pulse oximeter, is it a free application? Does it work with any operating system? Is it compatible with other pulse ox devices?**

Pulse oximetry and respiratory rate were measured using two different but integrated applications. A 1-minute pulse oximetry spot check was performed with the LGT pulse oximeter connected directly to the Android tablet – LGT is cited in the text. They also have pulse oximeters that can connect to iOS (Apple devices). The pulse oximeter spot check in our data collection tool can also work with Nonin or Masimo pulse oximeters. The RRate application for measuring respiratory rate by tapping on the screen each time the child breaths. RRate is thoroughly described in the cited paper, citation 13. RRate is also a stand-alone free application available for Android or iOS.

  

- **If primary outcome was 24hrs admission, how were patients who were sent home and died accounted for in the analysis. I mean these patients were the false negative of the screening tool.**

This is an unfortunate limitation of the study. However, the mortality rate for patients sent home was very low (0.2%, N=2). This is unlikely to have impacted the result. For future studies, mortality, date and cause of death will be recorded so that we can alter the positive admission outcome to include those patients who were sent home and died within a predetermined time period (capture the false negatives). This is described in the manuscript:

*A significant limitation of this study was the use of admission as a surrogate for acuity. Need for hospital admission is difficult to assess and may not accurately reflect a state of critical illness in children. We accounted for this by defining a positive outcome as admission for at least 24 hours to filter out those non-critically ill cases. We also conducted a 14-day post-discharge follow up call to identify children inappropriately sent home and found that both mortality and readmission rates were minimal (* *Table 4* *) (Strengths and limitations, Paragraph 3).*

  

- **Did they do the 10-fold cross validation at both the feature selection and model prediction stages on the whole dataset or did they do a 70/30 data split to test their final model of an unseen 30% of the data?**

We used recursive feature elimination with 5 time repeated 10-fold cross validation to select features to include in the model. For internal validation, we also used 10-fold cross validation where 9/10 folds comprised the “training set” (used to build the model), and the remaining fold comprised the “validation set” (used to test the model). This process was repeated 10 times, with each of the 10 subsamples used once as the validation data. The 10-fold internal validation procedure was repeated 5 times and the results were pooled to produce a single estimate of model performance.

  

- **If the missing data was minimal (<3% missing per predictor), why did the authors do an imputation exercise?**

We still wanted to maximize the number of cases included in the analysis. We did not want to discard the valuable information that was collected in the other fields that was not missing.


** **

- **“Of these variables, data collected after emergency treatment had the highest AUC: 0.80 (95% CI 0.74 to 0.85).“ Isn’t this pretty intuitive. I mean whoever received emergent treatment (and thus they got their data collected after those emergent services were given) will get admitted. Thus, in my view, the clinical application of such a predictor variable is meaningless.**
**  **

We agree with you. This variable was not included in the final prediction model. Univariate analysis was conducted on all candidate predictors to assess individual associations with the outcome, but it would not be appropriate to include this variable in a clinical prediction model. For future studies, this should not be considered a candidate predictor variable for the reasons you mentioned above.

  

- **Table 4, mortality in discharged patients was 2 while the parenthesis says (0%). It is 0.2%. I think one has to be accurate with such percentages especially if it represents a grave, rare event.**

Reporting for values in table 4 have been updated accordingly.


** **

- **“to suggest that mHealth (use of mobile devices with software applications to provide health services and manage patient information) can be used to strengthen health systems31. The computing power and display capability of even the entry level smartphones in low resource settings can be used as platforms to implement clinical prediction models32. The digital platform also allows for real time monitoring of user performance and compliance and optimization of work flow.” This is something the authors mention in their introduction and even as a conclusion. Their current data is not enough to drive the conclusion around the utility of this model as an mobile app. Additionally they can’t claim superiority over WHO- ETAT, for that they have to really do a proper trial.**

We agree with you. We mention that the study represents a step forward in strengthening triage systems in LMICs by presenting a data-driven prediction model to be integrated into a real time electronic digital platform. The model needs to be implemented into a mobile app and externally validated in a variety of settings in low-and-middle-income countries to for any conclusions regarding model utility can be drawn, which we acknowledged in the limitations:

*Finally, the lack of external validity poses a significant limitation to this study. The model is currently being validated in an independent multi-site study that will include clinical implementation to assess performance in varied geographical locations, seasons, and with different disease prevalence and severity [35] (Strengths and limitations, Paragraph 7).*

 [35] Mawji A, Li E, Komugisha C, *et al.*: **Smart triage: triage and management of sepsis in children using the point-of-care Pediatric Rapid Sepsis Trigger (PRST) tool.** *BMC Health Serv Res. *2020; **20**(1): 493.

 We also modified the conclusion to reflect this:

*We developed a logistic triage model for rapid identification of critical illness in children at first contact. The triage model, comprised of five objectively measurable variables (transformed oxygen saturation, pulse rate, temperature, weight, MUAC) and three parent reported variables (lethargy, inability to drink/breastfeed, difficulty breathing) had good discrimination, calibration, and internal validation. The model can be easily integrated into a digital health platform and used with minimal clinical training. External validation is required prior to adoption (Conclusion).*

  

1. ** “In addition, pulse oximetry can be conducted with mobile device by attaching low-cost sensors to enable objective measurements and alleviate the need to perform manual data entry of values read from a separate monitor”. It is true but sometimes that cross talk between the device output and auto-populating data into another software may not be that easy of device company agnostic. The commercial value of such a software which facilitates this cross talk may not be affordable.**
**  **

We agree that this can be the case, but for this study it was not, and thus was an advantage of our data collection app. For this study, the pulse oximeter we used from LGT connected directly to the tablet and there was no intermediate software needed – the app itself contained the library to use the incoming values from the pulse oximeter. Stand-alone pulse oximeters that communicate with apps through Bluetooth are becoming more popular, but you are generally restricted to using the manufacturer’s app. Since we do not need to do this, it is an advantage and a cost-saving approach. We would also like to provide incentives for device manufacturers to add differentiators to their products. A algorithm could be added to any of the current stand alone devices without significant technical modification.

  

- **“The inclusion of RRate, an app for measure respiratory rate by tapping on the screen also enables faster collection of respiratory rate with less effort than counting breaths”. Again, company proprietorship and device agnostic cross talk may preclude its generalizability.**

RRate is a free application available for Android or iOS. Both versions are setup to provide their resulting respiratory rate values to other apps on the same device free of charge, in much the same way as many apps switch to the camera app and let you take a photo and then feed that photo back into the requesting app. This then takes some development work for the developer of the app but is done in a standard method and is free. These technical details are beyond the scope of this paper.

  

- **The greatest flaw in the model is that it excluded a very “serious” sign and symptoms like apnea, bleeding and new onset hemiparesis due to unavailability of data.**

These are rare events that are clear signs of emergency. The model was designed to guide frontline health workers in those identifying critically ill (or high risk) children for which there are no obvious indicators such as those mentioned above. We have addressed this in the manuscript:

*Some risk factors could not be used in multivariable analysis due to low prevalence in the study population. This may indicate need for a study with a larger sample size. When a single sign or symptom with low population prevalence, such as unconsciousness, is well known to indicate risk this should be used as a danger sign prior to the use of any risk prediction tool. Risk prediction within a mobile app is only necessary when multiple predictors are required to augment the prediction. Alternatively, if these danger signs are easy to assess and strongly correlated with admission, these predictors may be treated as independent triggers for admission. This cascade of decision rules can be readily implemented in a digital platform with the complexity hidden from the user (Strengths and limitations, Paragraph 5).*

**

# Peer review: Reviewer response for version 2

**DOI:** 10.21956/wellcomeopenres.18080.r41846

**Published:** 2021-01-26

**Authors:** Andrew Argent

**Affiliations:** 1 Department of Paediatrics and Child Health, University of Cape Town, Cape Town, South Africa

**General Comments**

 My thanks to the authors for the positive response to the review, the changes that have been made, and the additional data that has been provided.

**Specific Comments**

***Title***

 I wonder if the title is accurate in the use of the term “triage”. The study showed that certain features were associated with hospital admission (in children who were reviewed and entered into the study after initial clinical assessment and treatment).

***Introduction***

 Thanks for the modifications.

***Methods***

*Ethics*

 I accept that the data was not collected regarding the actual numbers of patients who refused consent. Unfortunately it does put a question mark over the extent to which patients and their families gave free consent to participate in this study. I think that this should be highlighted in the shortcomings of the study.

*Population*

 I think that the terminology of “outpatient department” is potentially confusing. In many settings the outpatient department will see patients who have booked appointments and re not presenting primarily for emergency treatment. Most “outpatient” departments are open only during normal working hours. What the authors are describing is a clinical area that sees all the patients presenting for assessment and therapy at all hours of the day and night, including weekends and public holidays. That would be described as an emergency service in many settings.

 If in fact the elective visits for ongoing care are seen in the same setting and by the same team that provides triage, emergency care and intervention, then that has to be expressed clearly. It also has significant implications for the population that is being screened.

 I am still not clear if trauma/burns/acute surgical emergencies were seen in the same area.

***Results***

 Thank you for the clarification of the data as requested.

 As regards the comment from me that: “It seems strange that the 3 children with “uncontrolled bleeding” and the 3 children with apnoea did not require admission. Both events would seem as if they were good reasons for admission. Similarly, it seems strange that 6 of the children with “newly onset hemiparesis” were not admitted to the hospital.” – I accept the authors response, but would be happier if there was some comment on these events in the discussion (not just in the response to me).

***Discussion***

 I wonder if it is worth highlighting (possibly also in the title), that this model seems to be accurate to differentiate between those who can be sent home and those who require hospital admission.  That is very different to a common discussion of triage, where the process is used to separate patients into groups with different priorities for acute medical attention.

Is the work clearly and accurately presented and does it cite the current literature?

Yes

If applicable, is the statistical analysis and its interpretation appropriate?

Yes

Are all the source data underlying the results available to ensure full reproducibility?

No

Is the study design appropriate and is the work technically sound?

Partly

Are the conclusions drawn adequately supported by the results?

Partly

Are sufficient details of methods and analysis provided to allow replication by others?

No

Reviewer Expertise:

I have experience in the evaluation of critically ill children for admission, particularly in areas of lower resource availability

I confirm that I have read this submission and believe that I have an appropriate level of expertise to confirm that it is of an acceptable scientific standard.

# Peer review: Reviewer response for version 1

**DOI:** 10.21956/wellcomeopenres.16810.r36229

**Published:** 2019-09-04

**Authors:** Andrew Argent

**Affiliations:** 1 Department of Paediatrics and Child Health, University of Cape Town, Cape Town, South Africa

General Comments

 In this study, data at the time of paediatric outpatient presentation was collected. The data was collected by a research nurse, with some of the information coming from parents, and some from patient records (when patients had been identified as being emergency patients by the clinical staff).

 The standard outcome for “triage” was whether the patient was admitted to hospital. There is unfortunately no information as to the outcomes for those patients including mortality, duration of hospital stay, interventions required etc.  

 It would also have been useful to have a sense of the fit across a range of different patients in different settings.

 Specific Comments

 Introduction

 Para 1

 I do understand that sepsis is a major cause of mortality for children, but this study does not provide information as to the diagnosis in the children who were admitted to hospital. The study is not specific to sepsis, and presumably involves a cohort of children with a wide range of diagnoses. In that context it does not seem entirely appropriate to focus so clearly on sepsis in the introductory material.

 I wonder if there are better references to support the statement that “triage systems in low-income settings continue to face challenges including limited numbers of expert clinicians and lack of adequately trained health workers”. The references supplied are 10 years or more old.

 Methods

 Ethics

 Was consent actually refused in any case? It would be useful to see this data.

 Population

 The outpatient department is quoted as serving approximately 20 000 children per annum.  During the 6-month period approximately 10 000 children were seen, suggesting that the clinic was open only for the study hours (Monday to Friday, 8am to 5pm).  Firstly, do all emergency patients (children) present to the outpatient department, or are there other routes of presentation? Secondly what happens to children who present as emergencies outside of those hours?

 I am not sure of the qualifications and experience of “two or three clinicians”.  Are these doctors, nurses, clinical assistants, or any other category of staff?

 Eligibility

 It is not clear from this statement as to whether children with trauma, burns, or acute surgical conditions would have been seen in the paediatric OPD. It does seem from the table of candidate predictor variables (Table 1) that trauma patients were included, but it would be useful to clarify this, as in many settings trauma patients are not seen by the paediatric teams.

 Study procedures

 It seems that children who did require emergency treatment would only have been seen by the study nurse after receiving therapy, and so the data collected would not have been the presenting findings. To what extent could the results have been affected by this (particularly as 86% of the admitted participants were seen after emergency treatment)?

 The oxygen saturation was presumably measured while in room air (that is not specifically stated in the methods), but that information seems essential to the estimation of the transformed saturation.

 To what extent could the data regarding saturation have been affected by the fact that Nairobi is at nearly 6000ft above sea level?

 Outcome

 The outcome recorded was admission to hospital for 24 hours or longer. Unfortunately, that means that children who were inappropriately sent home (and perhaps died or sought help elsewhere) were not picked up in the study. It also means that there is no differentiation between an acute illness that needs urgent intervention and a more chronic illness that requires admission and investigation (but possibly not immediate life-saving therapy).

 Results

 It would have been useful to have a sense of the age-spread of the study population, and the profile of reasons for admission.

 It is demonstrated that 35 patients had missing data for oxygen saturation and heart rate. Was the missing data primarily in the group who were admitted, or was it evenly distributed across the groups?  Clearly one is concerned that saturation data may be more difficult to achieve in very sick children.

 It seems strange that the 3 children with “uncontrolled bleeding” and the 3 children with apnoea did not require admission. Both events would seem as if they were good reasons for admission.  Similarly, it seems strange that 6 of the children with “newly onset hemiparesis” were not admitted to the hospital.

 Discussion

 As highlighted by the authors a major limitation of the study is that hospital admission was used as a surrogate for acuity of illness. In addition, there was probably overlap between the criteria used by the research team and those used by the clinical team which may have biased the study.

 It does seem to make sense to exclude rare events from the triage data collection, but as a category “rare events” may be a strong indication for admission.  As an example it would seem likely that a child with a large tumour would be admitted to hospital regardless of the overall triage score.

 Conclusions

 The model has been shown to conform with the rate of patient admission. That has potential, but as the authors point out, it will require validation in other settings. It will also need to be applied prospectively to patients coming through the system to see how well it actually predicts in another set of patients in the same setting.

Is the work clearly and accurately presented and does it cite the current literature?

Yes

If applicable, is the statistical analysis and its interpretation appropriate?

Yes

Are all the source data underlying the results available to ensure full reproducibility?

No

Is the study design appropriate and is the work technically sound?

Partly

Are the conclusions drawn adequately supported by the results?

Partly

Are sufficient details of methods and analysis provided to allow replication by others?

No

Reviewer Expertise:

I have experience in the evaluation of critically ill children for admission, particularly in areas of lower resource availability

I confirm that I have read this submission and believe that I have an appropriate level of expertise to state that I do not consider it to be of an acceptable scientific standard, for reasons outlined above.

# Response

**Published:** 2020-12-02

**Authors:** Alishah M

**General Comments**

**In this study, data at the time of paediatric outpatient presentation was collected. The data was collected by a research nurse, with some of the information coming from parents, and some from patient records (when patients had been identified as being emergency patients by the clinical staff).**

****

**The standard outcome for “triage” was whether the patient was admitted to hospital. There is unfortunately no information as to the outcomes for those patients including mortality, duration of hospital stay, interventions required etc.**

 We would agree that information on mortality, duration of hospital stay, and interventions would also be valuable outcome indicators. The resources available for the study did limit our ability to collect a comprehensive set of outcome information. While we did capture mortality, the number of deaths was minimal over the 6-month study duration (N=6) and thus we could not conduct meaningful mortality analysis (insufficient outcomes). We chose admission status, defined as “children admitted >24 hours”, as we believe that this should also predict who might need community referral for admission. We have included information on mortality and duration of stay in the updated version of the manuscript.

**It would also have been useful to have a sense of the fit across a range of different patients in different settings.**

 We agree with the reviewer. This is mentioned in the strengths and limitations section. Future ongoing research is assessing the implementation and performance assessment of the model in varied geographical locations, seasons, and with different disease prevalence and severity.

**Specific Comments**

**Introduction**

**Para 1**

**I do understand that sepsis is a major cause of mortality for children, but this study does not provide information as to the diagnosis in the children who were admitted to hospital. The study is not specific to sepsis, and presumably involves a cohort of children with a wide range of diagnoses. In that context it does not seem entirely appropriate to focus so clearly on sepsis in the introductory material.**

 This study involves a cohort of children with a wide range of infectious diseases. We agree that this study is not specific to sepsis, but rather a wide range of infectious diseases that can lead to sepsis if not diagnosed and treated in a timely manner. We have clarified in the introduction section of the updated manuscript that *“The model can be used to guide frontline health workers in early identification of children at risk of developing severe infections, including sepsis so that lifesaving treatment can be instituted.”*

****

**I wonder if there are better references to support the statement that “triage systems in low-income settings continue to face challenges including limited numbers of expert clinicians and lack of adequately trained health workers”. The references supplied are 10 years or more old.**

 We have included a newer reference in the updated manuscript.

 Hategeka C, Mwai L, Tuyisenge L. Implementing the Emergency Triage, Assessment and Treatment plus admission care (ETAT+) clinical practice guidelines to improve quality of hospital care in Rwandan district hospitals: healthcare workers’ perspectives on relevance and challenges. BMC Health Serv Res. 2017;17(1):256–12

**Methods**

**Ethics**

**Was consent actually refused in any case? It would be useful to see this data.**

 Unfortunately, we did not collect data on refused cases. We will keep this in mind for future studies.  

****

**Population**

**The outpatient department is quoted as serving approximately 20 000 children per annum.  During the 6-month period approximately 10 000 children were seen, suggesting that the clinic was open only for the study hours (Monday to Friday, 8am to 5pm).  Firstly, do all emergency patients (children) present to the outpatient department, or are there other routes of presentation? Secondly what happens to children who present as emergencies outside of those hours?**

 The manuscript states that the OPD is serves **over** 20,000 children per year. This was an estimate based on the last couple years that was provided to us by the hospital. All children present to the OPD, including those emergency patients seen outside study hours. There are significantly fewer patients presenting to the OPD between 5pm – 6am as well as on weekends.

**I am not sure of the qualifications and experience of “two or three clinicians”.  Are these doctors, nurses, clinical assistants, or any other category of staff?**

 This refers to physicians. I have clarified this in the updated version of the manuscript.

**Eligibility**

**It is not clear from this statement as to whether children with trauma, burns, or acute surgical conditions would have been seen in the paediatric OPD. It does seem from the table of candidate predictor variables (Table 1) that trauma patients were included, but it would be useful to clarify this, as in many settings’ trauma patients are not seen by the paediatric teams.**

 At Mbagathi Hospital, all paediatric patients are initially seen at the pediatric OPD. I have clarified the eligibility criteria in the updated version of the manuscript:

*“Children aged 2–60 months seeking treatment for an acute illness at Mbagathi hospital on weekdays between 8:00 am and 5:00 pm were eligible for enrollment. Patients coming for elective procedures, such as elective surgery or for cardiac follow up, were excluded from the study. Patients presenting for elective care or treatment for chronic illnesses were excluded from the study.”*

**Study procedures**

**It seems that children who did require emergency treatment would only have been seen by the study nurse after receiving therapy, and so the data collected would not have been the presenting findings. To what extent could the results have been affected by this (particularly as 86% of the admitted participants were seen after emergency treatment)?**

 We do appreciate that data collection after initiation of emergency treatment may have affected the results. However, patient safety is the first priority. We did not want study procedures to delay or interfere with clinical care to any patient and so we had to wait until emergency treatment was initiated and the patient was stable before collecting data. We did try to minimize the effect of this by prioritizing collection of variables that are subject to change (vitals, danger signs). The need for emergency treatment is indicative of a serious illness. It would thus make sense that many of the admitted cases were children who were seen after emergency treatment.

**The oxygen saturation was presumably measured while in room air (that is not specifically stated in the methods), but that information seems essential to the estimation of the transformed saturation. To what extent could the data regarding saturation have been affected by the fact that Nairobi is at nearly 6000ft above sea level?**

 Data on oxygen saturation could certainly be affected by the altitude of Nairobi. In future studies, we will use an altitude adjusted saturation gap formula to account for this:

 Zhou A, Karlen G, Brant W, et al.: The saturation gap: A simple transformation of oxygen saturation using virtual shunt. *bioRxiv.* 2018;3–10. 10.1101/391292

**Outcome**

**The outcome recorded was admission to hospital for 24 hours or longer. Unfortunately, that means that children who were inappropriately sent home (and perhaps died or sought help elsewhere) were not picked up in the study. It also means that there is no differentiation between an acute illness that needs urgent intervention and a more chronic illness that requires admission and investigation (but possibly not immediate life-saving therapy).**

 The eligibility criteria actually included only children with acute illnesses who presented to the OPD. Children with chronic illnesses, or patients coming for elective procedures and follow up were not eligible for this study. I have clarified this in the updated version of the manuscript:

*“Children aged 2–60 months seeking treatment for an acute illness at Mbagathi hospital on weekdays between 8:00 am and 5:00 pm were eligible for enrollment. Patients coming for elective procedures, such as elective surgery or for cardiac follow up, were excluded from the study. Patients presenting for elective care or treatment for chronic illnesses were excluded from the study.”*

 We also conducted a phone interview 14 days post-discharge for those participants who were not initially admitted to the hospital to determine mortality status, children returning to the hospital with the same illness, and children admitted to a hospital (the study hospital or elsewhere) for the same illness. This was to capture those children who were inappropriately sent home. I have included this information in the updated manuscript:

*“A telephone interview was conducted post-discharge to determine 1) mortality status, 2) whether the child completely recovered from the illness, 3) if the child returned to a hospital or health center seeking help for the same illness, 4) if the child was admitted to a hospital or health center for the same illness.”*

*“Taking into consideration that children could be inappropriately sent home, we captured mortality and admission to a hospital or other health facility after being sent home using a telephone interview 14 days following discharge. Among discharged participants, mortality (0.2%) and re-admission (2%) rates were minimal (Table 4).”*

**Results**

**It would have been useful to have a sense of the age-spread of the study population, and the profile of reasons for admission.**

 I have included this in the updated version of the manuscript (see Tables 3 and 4).

**It is demonstrated that 35 patients had missing data for oxygen saturation and heart rate. Was the missing data primarily in the group who were admitted, or was it evenly distributed across the groups?  Clearly one is concerned that saturation data may be more difficult to achieve in very sick children.**

 Both the admitted (N = 4 missing) and not admitted (N = 31 missing) groups had an even distribution of missing data for oxygen saturation (~3% missing).

**It seems strange that the 3 children with “uncontrolled bleeding” and the 3 children with apnoea did not require admission. Both events would seem as if they were good reasons for admission.  Similarly, it seems strange that 6 of the children with “newly onset hemiparesis” were not admitted to the hospital.**

 We would strongly agree with these observations. We are unable to verify this information. It is also possible that some of these may have been data entry errors.

****

**Discussion**

**As highlighted by the authors a major limitation of the study is that hospital admission was used as a surrogate for acuity of illness. In addition, there was probably overlap between the criteria used by the research team and those used by the clinical team which may have biased the study.**

 We have explained above the motivation to use hospital admission as a surrogate for triage category. Due to the nature of this study, the overlap is inevitable and would be present in any study exploring predictors of critical illness in children. This is an ecological study in a clinical setting, so of course there will be bias, we agree and have acknowledged this. 

****

**It does seem to make sense to exclude rare events from the triage data collection, but as a category “rare events” may be a strong indication for admission.  As an example, it would seem likely that a child with a large tumour would be admitted to hospital regardless of the overall triage score.**

 In the discussion, we do mention how these rare events can be used as individual triggers for admission prior to the use of any risk prediction tool. The prediction tool will be most useful in the cases where the need for admission is not obvious. It is not realist to model these rare outcomes.

**Conclusions**

**The model has been shown to conform with the rate of patient admission. That has potential, but as the authors point out, it will require validation in other settings. It will also need to be applied prospectively to patients coming through the system to see how well it actually predicts in another set of patients in the same setting.**

 The model is currently being validated in an independent multi-site study that will include clinical implementation to assess performance in varied geographical locations, seasons, and with different disease prevalence and severity.
